# A comparison of the quality of integrated case formulations produced by UK psychiatric trainees and an artificial intelligence-assisted application

**DOI:** 10.1192/bjb.2025.10160

**Published:** 2026-06

**Authors:** Mohammed J. Abbas, Hannah Fosker, Harry Dudson, Simran Ramewal

**Affiliations:** 1 Leicestershire Partnership NHS Trust, Leicester, UK; 2 University of Leicester, UK

**Keywords:** Artificial intelligence, machine learning, case formulation

## Abstract

**Aims and method:**

This study aimed to evaluate an artificial intelligence-assisted tool for psychiatric case formulation compared with human trainees. Twenty trainees and an artificial intelligence system produced formulations for three simulated psychiatric cases. Formulations were scored using the integrated case formulation scale (ICFS), assessing content, integration and total quality. Time taken was recorded, and assessor predictions of formulation origin were analysed.

**Results:**

Artificial intelligence produced formulations significantly faster (<10 s) than trainees (mean 52.1 min). Trainees achieved higher ICFS total scores (mean difference 8.3, *P* < 0.001), driven by superior content scores, while integration scores were comparable. The assessor identified artificial intelligence-generated formulations with 71.4% sensitivity, but overall accuracy of who produced the formulations was only 58.3%.

**Clinical implications:**

Artificial intelligence shows promise as a time-saving adjunct in psychiatric training and practice, but requires improvements in generating detailed content. Optimising teaching methods for trainees and refining artificial intelligence systems can enhance the integration of artificial intelligence into clinical workflows.

Recent developments in artificial intelligence have created a huge paradigm shift by the automation of intellectual tasks, decision-making and creative processes. Artificial intelligence, as part of the so-called ‘fourth industrial revolution’, has led to ‘blurring the lines between the physical, digital, and biological spheres’, redefining how we live, work and relate to one another.^1^ Medicine is not excluded from these advancements. From diagnostic algorithms in radiology to personalised treatment plans in oncology, artificial intelligence is transforming healthcare by providing faster, more accurate and scalable solutions.^2^ The UK National Health Service (NHS) Long Term Plan emphasised that the adoption of artificial intelligence could enhance diagnostic accuracy, personalise treatments and improve patient outcomes.^3^

In psychiatry, a field characterised by its complexity and reliance on subjective clinical judgement, artificial intelligence applications are emerging as tools to augment clinical decision-making, predict treatment outcomes and even analyse patient narratives for diagnostic purposes.^4–7^ Ethical concerns related to the use of artificial intelligence in healthcare have been highlighted.^4,7^

Case formulation in psychiatry is one of the core competencies that psychiatrists are expected to master.^8^ However, previous studies show that case formulations are either almost absent from assessment letters^9^ or are of poor quality.^10,11^

The integrated case formulation (ICF) model^12^ was developed as an attempt to integrate the different aspects of the psychiatric history to produce a narrative, integrated and structured summary.

Recent advancements in machine learning have enabled artificial intelligence systems to analyse and summarise complex text, making them suitable candidates for assisting in case formulation. Artificial intelligence tools could potentially address the time and training barriers by providing structured formulations for review and refinement by clinicians. However, the quality and reliability of such tools in replicating human expertise remain critical areas of investigation. This study aims to evaluate an artificial intelligence-assisted tool for psychiatric case formulation, comparing its performance with that of trainees and trust-grade doctors, and exploring its potential role in education and clinical practice. In addition, we explored whether doctors’ gender, grade or previous teaching in ICF have had any impact on that comparison.

## Method

Psychiatric trainees and trust-grade doctors working in Leicestershire Partnership NHS Trust (LPT) were approached by email or a trainees’ WhatsApp group and invited to participate in this study. Those who agreed to participate were divided into three groups, using stratified randomisation to ensure equal distribution of the different grades. Trainees were then sent an email that included a guide to the ICF model with an example of a case formulation. They were also sent the psychiatric history and mental state examination of the case allocated to their group. They were then asked to produce a case formulation, and to record the time taken to produce it (in minutes). In addition, they were asked to report their gender, grade and whether they had had any teaching on the ICF model.

The scripting language Python was used (by M.J.A.) to create a web application that can submit queries to ChatGPT-3.5 Turbo, using a unique OpenAI application programming interface key (OpenAI, San Francisco, CA, USA; see https://openai.com/index/gpt-3-5-turbo-fine-tuning-and-api-updates/). PyCharm version 2024.2.1 Community Edition for Windows (JetBrains, Amsterdam, The Netherlands; see https://www.jetbrains.com/pycharm/) was used to create the app. When executed, a webpage is created in a local host. The query to OpenAI consisted of a case history and mental state examination, the same guide and instructions given to trainees regarding production of an ICF. There was no further specific training of the model in relation to producing the ICF.

Three simulation psychiatric cases (case 1: borderline personality disorder; case 2: bipolar affective disorder; and case 3: obsessive–compulsive disorder) were developed by the authors. Each case contained a detailed psychiatric history and mental state examination. For each case, three case formulations were produced by the artificial intelligence, making a total of nine.

Case formulations produced by trainees or artificial intelligence were anonymised, each evaluated using the ICFS.^^12^^ Scoring was carried out by a consultant psychiatrist (H.F.) who is familiar with the ICF model and has used it for many years. The assessor read the case histories before scoring the case formulations using the ICFS.^12^ The ICFS is an 18-item scale, 12 of which cover the content and 6 the integration. Each item is scored as either 0 (not covered or extremely unclear, or not plausible/relevant), 1 (covered partially: more detail needed or unclear) or 2 (covered fully: relevant details included, clear, understandable, plausible/relevant). Three scores were produced: ICFS content (maximum score 24), ICFS integration (maximum score 12) and total score (maximum score 36). In addition, the assessor was asked to provide a response to ‘I believe this was written by AI’, using a Likert scale ranging from 1 (strongly disagree) to 5 (strongly agree). The time required for formulation creation was recorded. The assessor, blinded to the source of the formulations, scored all entries and predicted whether each formulation had been generated by a trainee/trust-grade doctor or artificial intelligence. The predictions were analysed to assess their accuracy, sensitivity and specificity.

Descriptive statistics (mean, standard deviation) were calculated for ICFS scores and time, and were compared using *t*-tests or analysis of variation. Gender, grade and teaching history were analysed for differences in ICFS total scores using similar tests. Assessor performance in predicting formulation source was evaluated with a confusion matrix. To produce a binary outcome, the responses strongly disagree and disagree were combined into one category, and the same was done for ‘strongly agree’ and ‘agree’. When the response was neutral (case 3), this was excluded from the analysis. Statistical analysis was performed using SPSS. The project was approved by the Quality Improvement Design Huddle of LPT.

## Results

Twenty trainees and trust-grade doctors (9 male, 45% and 11 female, 55%) returned completed case formulations. Their grades were Core Trainees (10, 50%), Speciality Trainees (5, 25%) and Foundation Year/trust-grade doctors (5, 25%).

Artificial intelligence performed significantly better in regard to the amount of time (<10 s) needed to produce ICFS compared with trainees, who needed an average of 52.1 min (s.d. 19.4). This difference (95% CI 38.6–65.4) was statistically significant, *t*(19) = 12, *P* ≤ 0.001; see Table 1).

**Table 1:** Integrated case formulation scale (ICFS) scores and time taken to produce formulation by artificial intelligence (AI) and trainees

Cases	AI/trainees	Time (min)		ICFS content		ICFS integration		ICFS total	
		Mean	s.d.	Mean	s.d.	Mean	s.d.	Mean	s.d.
All cases	Trainees	52.1	19.4	21.3	2.2	8.8	2.5	30.2	4.0
	AI	0.07	0.4	15.0	2.1	6.9	2.1	21.9	4.0
	MD and CI	52.0 (38.6 to 65.4)^***^		6.3 (4.5 to 8.2)^**^		1.9 (−0.2 to 4.1)		8.31 (4.9 to 11.6)^**^	
Case 1	Trainees	48.7	18.5	19.7	2.9	7.5	3.2	27.2	5.1
	AI	0.07	0.01	14.7	3.2	7.3	2.1	22.0	2.6
	MD and CI	48.6 (22.5 to 74.6)^***^		5.00 (−0.06 to 10.06)		0.17 (−4.65 to 4.99)		5.17 (−2.39 to 12.72)	
Case 2	Trainees	49	21.7	22.0	1.5	9.0	2.4	31.0	3.2
	AI	0.08	0.01	15.7	2.1	6.7	4.7	22.3	6.8
	MD and CI	48.9 (19.6 to 78.2)^***^		6.3 (3.8 to 8.8)^***^		2.3 (−2.4 to 7.1)		8.7 (2.2 to 15.2)^**^	
Case 3	Trainees	59.7	18.2	22.2	1.5	10.0	1.5	32.2	2.3
	AI	0.06	0.01	14.7	1.5	6.7	2.1	21.3	3.5
	MD and CI	59.6 (33.9 to 85.3)^***^		7.50 (5.0 to 9.9)^***^		3.3 (0.5 to 6.2)		10.8 (6.3 to 15.4)^***^	
Gender	Female	49.3	15.9	22.2	1.5	9.8	2.2	32.0	3.0
	Male	55.6	23.5	20.3	2.6	7.7	2.5	28.0	4.2
	MD and CI	6.3 (−12.3 to 24.8)		1.8 (3.8 to 0.1)		2.1 (4.4 to 0.1)		4 (7.4 to 0.6)*	
Grade	CT	45.7	16.3	21.6	2.6	9.0	2.4	30.6	4.0
	FY/trust grade	63.0	17.5	20.6	2.1	9.0	3.2	29.6	5.1
	ST	54.0	24.9	21.6	1.8	8.4	2.7	30.0	3.7
Had teaching	Yes	50.8	19.2	20.8	2.5	7.8	2.5	28.6	3.9
	No	54.6	21.1	22.4	1.1	10.7	1.1	33.1	2.0
	MD and CI	−3.8 (−23.3 to 15.7)		−1.66 (−3.8 to 0.4)		−2.9 (−5.0 to −0.7)*		−4.5 (−7.9 to −1.1)*	

MD, mean difference; CT, core trainees; FY, foundation year; ST, speciality trainees.

*P < 0.05, **P < 0.01, ***P < 0.001.

**Fig. 1:**
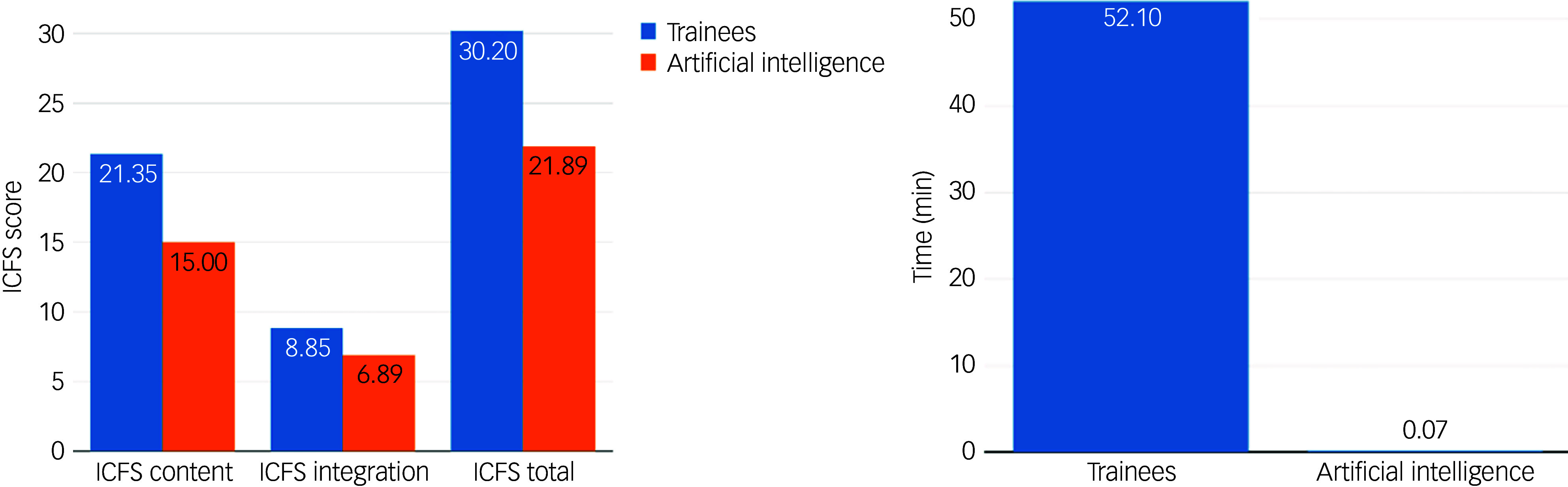
Integrated case formulation scale (ICFS) scores and time taken to produce the formulations.

The total scores for ICFS were significantly higher in trainees compared with artificial intelligence (mean difference 8.3, 95% CI 4.9–11.6, *t*(27) = 5.12, *P* ≤ 0.001). This difference is largely due to the better performance of trainees on the content part of ICFS (mean difference 6.3, 95% CI 4.5–8.2, *t*(27) = 7.19, *P* ≤ 0.001). There was no statistically significant difference in the scores of the two groups in the ICFS integration subscale. This pattern was consistent for cases 2 and 3 but not for case 1, where there were no statistically significant differences between the scores of trainees and artificial intelligence (Table 1 and Fig. 1). Formulations produced by artificial intelligence included correct diagnoses and did not include factual errors or additional information. Box 1 shows an example of a formulation produced by artificial intelligence.

Box 1 An example of integrated case formulation (ICF) produced by artificial intelligence.

Adam Johnson is a 35-year-old man, currently unemployed and receiving benefits, with a diagnosis of bipolar affective disorder. Family history reveals a genetic vulnerability to mental illness, with his brother also diagnosed with bipolar affective disorder and his father exhibiting maladaptive personality traits. Pregnancy, birth and development were unremarkable. His early life experiences were marked by a critical and distant father, leading to long-standing low self-esteem and sensitivity to criticism.

These experiences have contributed to his persistent fear of negative evaluation, avoidance of others and an anxious personality trait. Despite these challenges, Adam managed to achieve academically, obtaining nine GCSEs and three A-levels. However, his career as a journalist was interrupted by a severe manic episode at age 28. Adam’s current presentation includes a severe manic episode with psychotic features, triggered by stopping his medication and resuming cannabis use. He exhibits manic symptoms such as elevated mood, irritability, grandiosity and impulsivity, along with psychotic symptoms including paranoid delusions and auditory hallucinations. Psychological stressors, including relationship issues and substance use, have triggered and maintained his current episode. His premorbid personality traits, such as emotional reactivity, impulsivity and sensitivity to criticism, suggest underlying vulnerabilities contributing to his illness.

In the past, Adam had episodes of major depressive disorder and bipolar affective disorder, with multiple hospital admissions. His current episode required assessment by the crisis team and resulted in his detention under Section 2 of the Mental Health Act. These recurrent episodes have impacted his functioning, leading to periods of unemployment and reliance on benefits. While Adam receives support from his mother and maintains a small but reliable social network, his current relationship and sporadic contact with his child demonstrate ongoing interpersonal challenges. Adam’s mental state examination reveals a dishevelled appearance, rapid and pressured speech, labile mood and incongruent affect. His thought process is tangential, with racing thoughts and fluctuating grandiose and suicidal ideas. Insight is limited during manic episodes but moderate otherwise. The impact of his current episode on functioning is evident in his unemployment, social withdrawal and the need for intensive psychiatric care. The diagnosis for Adam Johnson is bipolar affective disorder, currently presenting with a manic episode with psychotic features. The episode has been triggered and maintained by psychosocial stressors, non-adherence to medication and substance use. Adam’s premorbid personality traits, including emotional reactivity and impulsivity, along with his interpersonal difficulties and limited social support, contribute to the complexity of his presentation. Treatment should focus on stabilising the current episode, addressing substance use, enhancing social support and improving insight and adherence to medication to prevent future relapses.

Two formulations produced by trainees included additional, inaccurate details not found in the case histories. One was a minor assumption on the rationale behind changing medication, while the other included an additional medication not mentioned in the history.

Female trainees scored higher on ICFS total scores (mean difference 4.0, 95% CI 7.4–0.6, *t*(18) = −2.49, *P* = 0.023) but not on content or integration scores separately, nor on time. Grade had no significant impact on ICFS scores. Those who had been provided teaching performed worse than those who had not on both integration (mean difference −2.9, 95% CI −5.0 to −0.7, *t*(18) = −2.81, *P* = 0.012) and total scores (mean difference −4.5, 95% CI −7.9 to −1.1, *t*(18) = −2.79, *P* = 0.012).

The confusion matrix for assessor predictions of the origin of formulations is presented in Table 2. This shows that, out of the 7 cases produced by artificial intelligence, the assessor was able to predict 5 of them (sensitivity 71.4%); however, out of the 17 formulations produced by trainees, only 9 were predicted accurately (specificity 52.9%). The accuracy rate was 58.3%.

**Table 2:** Classification matrix

Cohort	AI/trainees	Prediction		
		Trainees	AI	Total
Group	Trainees	9	8	17
	AI	2	5	7
	Total	11	13	24

AI, artificial intelligence.

## Discussion

To the best of our knowledge, this is the first study to compare the quality and efficiency of psychiatric formulations produced by ChatGPT and those produced by psychiatric trainees of different grades. The formulations were based on the ICF model, which itself is based on psychiatric history. A previous study used ChatGPT to generate psychodynamic case formulations, and the results were rated as appropriate.^13^ The artificial intelligence model we used, ChatGPT, was given very basic instructions on how to produce case formulation, without any training. In spite of this, the model was able to produce case formulations within a negligible amount of time.

### Quality of case formulation

The quality of the formulations produced by the application was as good as that produced by trainees in the domain of integrating information, as measured by the ICFS integration subscale. However, trainees performed better in covering more information as measured by the ICFS content subscale. Because of this difference, total ICSF scores were better for trainees compared with artificial intelligence. Formulations produced by artificial intelligence did not introduce any incorrect new information and the diagnoses were all correct. Interestingly, two formulations produced by trainees included additional, inaccurate details not found in the case histories. This highlights that the risk of introducing inaccuracies is not exclusive to artificial intelligence: human formulations can also contain errors. The difference in ICFS content scores may reflect the possibility that specific programming of ChatGPT-3.5 Turbo could have led to a more condensed and generalisable output. While this trait enhances efficiency, it may limit the richness and specificity required for high-quality case formulations. Future iterations of artificial intelligence tools could address this by incorporating mechanisms to expand on key points and provide greater detail. It is of note that there was no statistically significant difference between trainees and artificial intelligence in one of the cases. Hence, it is possible that the observed difference between trainees and artificial intelligences could vary depending on the complexity of each case.

### Gender, grade and teaching

Female trainees outperformed their male counterparts on ICFS total scores, something that needs further exploration. Intriguingly, trainees with prior teaching on ICF performed worse than those without, particularly in regard to integration and total scores. This is surprising, because previous evidence showed that teaching case formulation in general^10,14^ and, more specifically, ICF,^12^ has led to improvements compared with no teaching. This unexpected finding may reflect over-reliance on structured teaching at the expense of experiential learning or the potential limitations of the teaching methods employed. Further investigation is needed to explore this paradox, and to optimise training approaches

The absence of significant differences in performance according to trainee grade raises important questions about the role of experience and training in mastering case formulation skills. It is possible that current training practices may provide a uniform foundation for developing these skills, irrespective of grade. However, it could also indicate a need for more advanced or targeted training at higher levels to ensure continued skill development and differentiation. Exploring the effectiveness of tailored educational interventions across grades could provide further clarity.

### Efficiency

The ability of the artificial intelligence tool to produce formulations in less than 10 s significantly outperformed trainees, who required an average of 52.1 min. This efficiency highlights the potential of artificial intelligence as a time-saving adjunct in clinical practice, particularly in environments with high workloads and time constraints. Taking into account the difference in quality, artificial intelligence tools could be utilised to provide preliminary drafts that clinicians can refine, thereby saving time while maintaining quality. Practical constraints and ethical issues would first need to be addressed, however.

### Assessor predictions and artificial intelligence sophistication

The assessor was able to correctly identify 71.4% of artificial intelligence-generated formulations but could distinguish trainee formulations with only 52.9% specificity, resulting in an overall accuracy of 58.33%. While this indicates that artificial intelligence outputs are still somewhat recognisable, the narrowing gap between human- and machine-authored content highlights a growing challenge. This raises important ethical considerations, particularly around transparency and authorship when artificial intelligence-assisted tools are used. The use of artificial intelligence in such settings should be made very clear.

### Ethical issues

The use of artificial intelligence in psychiatric practice raises many ethical issues. Concerns about transparency, data security, privacy and bias have been outlined elsewhere.^15,16^ This has been highlighted in a recent publication by the UK Parliamentary Office of Science and Technology.^16^ Another pressing issue is the potential for clinician deskilling. Over-reliance on artificial intelligence tools may gradually reduce the development and application of critical thinking skills in health professionals, especially if artificial intelligence-generated formulations are accepted uncritically. This is particularly relevant in psychiatry, where narrative nuance and integration of biopsychosocial factors require expert judgement that may not be replicable by current artificial intelligence.

### Limitations

This study has certain limitations. The sample size was relatively small, particularly for subgroup analyses. Only one large language model (ChatGPT-3.5 Turbo) was used, limiting the generalisability of findings to other artificial intelligence systems. The model was used ‘out of the box’ without fine-tuning or advanced prompt engineering, which may have constrained its potential performance. The study relied on only three simulated psychiatric cases, which may not have captured the full range of complexity encountered in clinical practice. All formulations were scored by a single assessor, which, while ensuring consistency, introduces the risk of subjective bias and limits interrater reliability. Finally, asking the same assessor to both score quality and guess the source of the formulation introduces the potential for cognitive bias.

### Implications and future directions

The study highlights the potential of artificial intelligence-based tools as a valuable adjunct in psychiatric training and practice. The significant time savings demonstrated by the artificial intelligence system can alleviate clinician workloads by generating preliminary case formulation drafts, allowing clinicians to focus their efforts on reviewing and refining content. However, the results also highlight areas for development through further refinement in prompt design and model training. This could generate detailed and context-specific content, which remains a critical component of high-quality case formulations. Future research should explore the use of tailored data-sets, domain-specific fine-tuning and advanced prompting strategies. Comparative studies involving multiple artificial intelligence models, more diverse case materials, larger samples and multiple assessors are also needed to validate and extend these findings. Finally, any integration of artificial intelligence into psychiatric workflows must be accompanied by clear ethical guidelines that ensure transparency, maintain clinician accountability and safeguard against over-reliance or deskilling.

## Data Availability

The data that support the findings of this study are available on request from the corresponding author.

## About the authors

**Mohammed J. Abbas**, MBChB, MMed Sci, FRCPsych, is a consultant psychiatrist, Leicestershire Partnership NHS Trust, Leicester, UK and Honorary Associate Professor at the University of Leicester, Leicester, UK. **Hannah Fosker**, MRCPsych, is a consultant psychiatrist at Leicestershire Partnership NHS Trust, Leicester, UK. **Harry Dudson**, MBChB, is a Foundation Year 1 doctor at Leicestershire Partnership NHS Trust, Leicester, UK. **Simran Ramewal**, MBChB, is a core trainee in psychiatry, Leicestershire Partnership NHS Trust, Leicester, UK.

## References

[1] Schwab K. The Fourth Industrial Revolution. Portfolio Penguin, 2017.

[2] Topol EJ. Deep Medicine: How Artificial Intelligence Can Make Healthcare Human Again 1st ed. Basic Books, 2019.

[3] NHS England and NHS Improvement. Science in Healthcare: Delivering the NHS Long Term Plan. NHS England and NHS Improvement, 2020.

[4] Carr S. ‘AI gone mental’: engagement and ethics in data-driven technology for mental health. J Ment Health 2020; 29: 125–30. 32000544 10.1080/09638237.2020.1714011

[5] Hauser TU , Skvortsova V , De Choudhury M , Koutsouleris N. The promise of a model-based psychiatry: building computational models of mental ill health. Lancet Digit Health 2022; 4: e816–28. 36229345 10.1016/S2589-7500(22)00152-2 PMC9627546

[6] Hepdurgun C. The present and future of artificial intelligence applications in psychiatry. Noro Psikiyatr Ars 2024; 61: 1–2. 38496225 10.29399/npa.28725 PMC10943939

[7] Koutsouleris N , Hauser TU , Skvortsova V , De Choudhury M. From promise to practice: towards the realisation of AI-informed mental health care. Lancet Digit Health 2022; 4: e829–40. 36229346 10.1016/S2589-7500(22)00153-4

[8] Royal College of Psychiatrists. Core Psychiatry Royal College of Psychiatrists Core Training Curriculum (CT1–CT3). RCPsych, 2022.

[9] Abbas MJ , Premkumar L , Goodarzi A , Walton R. Lost in documentation: a study of case-formulation documentation in letters after outpatient assessment. Acad Psychiatry 2013; 37: 336–8. 24026375 10.1176/appi.ap.12060114

[10] McClain T , O’Sullivan PS , Clardy JA. Biopsychosocial formulation: recognizing educational shortcomings. Acad Psychiatry 2004; 28: 88–94. 15298859 10.1176/appi.ap.28.2.88

[11] Eells TD , Kendjelic EM , Lucas CP. What’s in a case formulation? Development and use of a content coding manual. J Psychother Pract Res 1998; 7: 144–53. 9527958 PMC3330487

[12] Abbas M , Walton R , Johnston A , Chikoore M. Evaluation of teaching an integrated case formulation approach on the quality of case formulations: randomised controlled trial. Psychiatrist 2012; 36: 140–5.

[13] Hwang G , Lee DY , Seol S , Jung J , Choi Y , Her ES , et al. Assessing the potential of ChatGPT for psychodynamic formulations in psychiatry: an exploratory study. Psychiatry Res 2024; 331: 115655. 38056130 10.1016/j.psychres.2023.115655

[14] Kendjelic EM , Eells TD. Generic psychotherapy case formulation training improves formulation quality. Psychotherapy (Chic) 2007; 44: 66–77. 22122169 10.1037/0033-3204.44.1.66

[15] McCradden M , Hui K , Buchman DZ. Evidence, ethics and the promise of artificial intelligence in psychiatry. J Med Ethics 2023; 49: 573–9. 36581457 10.1136/jme-2022-108447 PMC10423547

[16] Parliamentary Office of Science and Technology. Ethical Issues in Artificial Intelligence (POSTnote 738). UK Parliament, 2025.

